# Residual sleep disturbance and risk of relapse during the continuation/maintenance phase treatment of major depressive disorder with the selective serotonin reuptake inhibitor fluoxetine

**DOI:** 10.1186/1744-859X-9-10

**Published:** 2010-02-26

**Authors:** Huaiyu Yang, Lara Sinicropi-Yao, Sarah Chuzi, Soo Jeong Youn, Alisabet Clain, Lee Baer, Ying Chen, Patrick J McGrath, Maurizio Fava, George I Papakostas

**Affiliations:** 1 Depression Clinical and Research Program, Massachusetts General Hospital, Harvard Medical School, Boston, MA, USA; 2 Depression Evaluation Service Center, New York State Psychiatric Institute, Columbia University, New York, NY, USA

## Abstract

**Background:**

Relapse of major depressive disorder (MDD) is a common clinical problem. This study was designed to determine whether residual sleep disturbance (insomnia and hypersomnia) predict risk of relapse during the continuation and maintenance treatment of MDD.

**Methods:**

A total of 570 patients with MDD were treated with open-label, flexible dose fluoxetine (range 20 to 60 mg; mean dose = 45.8 mg/day; SD = 15.1) for 12 weeks. Under double blind conditions, 262 patients who achieved clinical response were randomly assigned to continue fluoxetine or to switch to placebo for 52 weeks or until relapse. Residual sleep disturbance during the baseline visit of the double-blind phase was assessed using items 4, 5, 6 (insomnia) and 22, 23, 24 (hypersomnia) of the Hamilton Depression Rating Scale (HDRS). Survival analysis was utilized to determine the effect of residual sleep disturbance on risk of relapse.

**Results:**

The severities of early (*P *> 0.05), middle (*P *> 0.05), late (*P *> 0.05), or total (*P *> 0.05) residual insomnia were not found to significantly predict risk of relapse during continuation and maintenance-phase treatment. Similarly, the severities of early bedtime (*P *> 0.05), oversleeping (*P *> 0.05), napping (*P *> 0.05), or total (*P *> 0.05) residual hypersomnia were not found to significantly predict risk of relapse during continuation and maintenance-phase treatment.

**Conclusion:**

The present study did not identify the severity of residual sleep disturbance among fluoxetine responders to predict risk of MDD relapse. The size of our sample may have precluded us from identifying more modest effects of residual sleep disturbance on the risk of relapse in MDD patients. Future studies are needed to further explore the relationship between residual sleep disturbance and relapse in MDD.

**Trial Registration:**

ClinicalTrials.gov Identifier: NCT00427128

## Background

Major depressive disorder (MDD) is a prevalent and, often recurrent illness that is associated with significant disability, morbidity, and mortality. MDD, according to the Diagnostic and Statistical Manual of Mental Disorders, fourth edition (DSM-IV) [1,2], is diagnosed by the presence of a constellation of symptoms including psychological (that is, sadness), behavioral (that is, suicidal gestures), cognitive (that is, concentration), and somatic/physical symptoms (that is, sleep, energy, psychomotor, and appetite disturbances). However, whether all depressive symptoms weigh equally with regards to their adverse impact on functioning, morbidity, mortality, and treatment outcome or whether some symptoms are more relevant than others remains, as of yet, undetermined. Furthermore, although the goal of treating MDD is to achieve full remission, it is common for many patients to continue suffering from residual symptoms after they respond to treatment [3]. Increasingly, researchers and clinicians have advocated the importance of treating residual symptoms and of exploring their neurobiological basis to develop better treatment options and to improve MDD outcome [4].

Several studies published to date suggest that sleep disturbance, namely insomnia and hypersomnia, may represent such symptoms that weigh more heavily with regards to their adverse impact on a number of outcomes. Specifically, a number of studies report an increased risk of subsequently developing MDD among non-depressed individuals complaining of insomnia (that is, insomnia may represent a prodromal symptom) [5,6]. Similarly, Roberts *et al*. [7] found that non-depressed individuals experiencing hypersomnia were at increased risk for developing MDD later on than individuals without hypersomnia. In addition, studies have established a positive correlation between the presence of sleep disturbance, including hypersomnia and insomnia, and a greater severity of depressive and anxiety symptoms [8,9], as well as increased suicide rates [10-12] among depressed patients. In addition, insomnia and hypersomnia appear to be among the most common residual symptoms following selective serotonin reuptake inhibitor (SSRI) treatment [13-15] and, often, require the use of specialized therapeutic interventions above and beyond the use of antidepressant monotherapy to ensure their full resolution [16-26]. Most importantly, there is preliminary evidence to suggest that residual sleep disturbance at remission may be especially deleterious with regards to its potential adverse impact on relapse/recurrence in MDD. Dombrovski and colleagues [27], for instance, used data from a clinical trial of maintenance treatment of late-life depression to analyze the impact of overall residual symptom levels as well as specific residual depressive symptom clusters on depressive recurrence. Both residual anxiety and residual sleep disturbance were found to be significant independent predictors of early recurrence across treatment groups. Identifying predictors of relapse in MDD is potentially clinically relevant, since such predictors could lead to the development of specialized treatment interventions which could reduce the risk of relapse/recurrence in MDD. Despite the encouraging results reported in the work by Dombrovski *et al*. [27], some studies have had different findings. For example, one Sequenced Treatment Alternatives to Relieve Depression (STAR*D) report [28] suggests that the association between residual sleep disturbance and MDD recurrence is far from being fully understood. For this reason, there is the need for more studies specifically examining the role of residual sleep disturbance as a predictor of relapse in MDD.

Therefore, the purpose of the present study was to explore the impact of residual sleep disturbance on the risk of relapse of MDD during the continuation/maintenance treatment with the SSRI fluoxetine among fluoxetine responders. In order to achieve this, we reanalyzed data from a 52-week, randomized, double-blind, placebo-controlled trial of fluoxetine continuation/maintenance treatment for MDD patients who had responded following a 12-week, open-label, flexible dose trial of fluoxetine. The original trial was specifically designed to identify predictors of relapse during the continuation/maintenance phase of MDD [29].

## Methods

The present work is a *post hoc *analysis of data from a clinical trial of fluoxetine in MDD [29]. For that trial, 627 patients, 18 to 65 years of age, with current MDD defined using DSM-IV criteria were recruited at one of two sites: either the New York State Psychiatric Institute in New York City, NY, USA (n = 372) or the Depression Clinical and Research Program of the Massachusetts General Hospital in Boston, MA, USA (n = 254). Institutional review boards at both sites approved the study, and all participants provided written informed consent. Diagnoses were made using the Structured Clinical Interview for DSM-IV Axis I Disorders, Patient Edition (SCID-PE) [2] with no minimum score for severity of depressive symptoms required for inclusion in the study.

Medical screening was performed, including medical history, physical examination, electrocardiogram (ECG), complete blood count (CBC), blood chemistry profile, thyroid function tests, urinalysis, and urine drug screen. Patients were excluded from the study if they were at significant risk of suicide; were pregnant or breastfeeding, were women not using effective contraception; had an unstable physical disorder; had a lifetime history of any organic mental disorder, psychotic disorder, or mania; had a history of seizures; had a neurological disorder that significantly affects central nervous system (CNS) function; had met criteria for substance abuse or dependence in the previous 6 months, other than nicotine dependence; were taking medications that may cause or exacerbate depression; had clinical or laboratory evidence of hypothyroidism without adequate and stable replacement therapy; or had a history of non-response to an adequate trial of a SSRI (defined as a 4-week trial of ≥ 40 mg of fluoxetine or the equivalent daily).

After a 1-week medication-free washout period, patients (n = 570) who continued to meet inclusion criteria and whose symptoms had not improved significantly (Clinical Global Impressions-Improvement (CGI-I) score >2) began a 12-week course of open-label treatment with fluoxetine. Patients were evaluated by a research psychiatrist weekly during the first 6 weeks, biweekly for the next 4 weeks, and weekly for the final 2 weeks. Target fluoxetine dosages were 10 mg/day for the first week, 20 mg/day for weeks 2 to 4, 40 mg/day for weeks 4 to 8, and 60 mg/day for weeks 5 to 12. The dose was increased to meet the target only if the patient tolerated the medication well, and it was increased to 40 mg daily for all patients who could tolerate it. Treatment response during the acute phase was defined as a CGI-I [30] scale score less than 3 for the last two visits of the open-label phase and no longer meeting DSM-IV criteria for MDD (by SCID-PE).

Patients who responded to the medication by week 12 (n = 262) entered a discontinuation phase during which they underwent random assignment, under double-blind conditions with computer-generated randomization, either to continue taking fluoxetine (n = 131) at the dose to which they had responded or to switch to placebo (n = 131) for 52 weeks or until relapse. Patients were seen monthly for the duration of the 52-week trial. By convention, the first 6 months of this period were considered the continuation phase, and the remainder, the maintenance phase. Identical fluoxetine or placebo capsules were dispensed by a research pharmacist. Compliance was monitored by counting returned capsules; participants whose adherence to the protocol was judged inadequate by the treating research psychiatrist were removed from the study. Relapse during the double-blind discontinuation phase was defined as having at least 2 consecutive weeks of ratings of less than 'much improved' on the CGI-I compared with ratings at entry into the study.

### Residual sleep disturbance measure

The Hamilton Depression Rating Scale (HDRS) [31] administered during the randomization visit (baseline visit of the continuation and maintenance phase of the study) was used to assess residual sleep disturbance. Specifically, HDRS items 4 (early insomnia, that is difficulty falling asleep), 5 (middle insomnia, that is awakenings during night-time), 6 (late insomnia, that is early morning awakenings) were utilized, 22 (early bedtime, that is falling asleep earlier than usual), 23 (oversleeping, that is waking up later than usual in the morning), and 24 (napping, that is daytime napping) were utilized. Each item is scored as 0, 1, or 2.

### Statistical tests

In order to test whether the presence of residual sleep disturbance predicted an increased risk of MDD relapse, we conducted a survival analysis (Cox proportional hazards regression), using SPSS 16.0 (SPSS Inc., Chicago, IL, USA), with time to relapse as the dependent variable and the following independent variables (1) the 17-item HDRS (HDRS-17) total score during the randomization visit (week 12), (2) gender, (3) chronicity (as defined in McGrath *et al*. [29]), (4) and each individual insomnia item score, each individual hypersomnia item score, total insomnia burden (the sum of items 4, 5, and 6), or total hypersomnia burden (the sum of items 22, 23, and 24) entered separately. Gender and chronicity were added to the model since they were found to predict risk of relapse (not differential by treatment) in the original study [29]. Two-sided statistical tests were employed, with alpha set at the 0.05 level of significance.

## Results

The results of the original study are reported elsewhere [29]. Briefly, the participants who underwent random assignment were a mean age of 38.2 years (SD = 10.9), and 55.3% were female. Their mean HDRS score was 17.1 (SD = 4.1) at baseline and 4.9 (SD = 3.1) at randomization; 22.7% of them had a history of dysthymia and thus currently had 'double depression'. About two-thirds (35%) of the participants had one or more comorbid axis I disorder, most commonly panic disorder (13.3%), social phobia (12.4%), and alcohol dependence (10.6%). During this phase, 85 participants left the study, on average 16.4 weeks (SD = 2.0) after randomization; 34 of them were from the placebo group (26.0% of the placebo group), and 51 were from the fluoxetine group (38.9%). The most common reasons for leaving during this phase were removal for inadequate adherence (30.6% of those who left the study), loss to follow-up (14.1%), and side effects (7.1%). Fluoxetine treatment during continuation and maintenance treatment was associated with continued remission (ratio of relapse hazard during placebo substitution to relapse hazard during fluoxetine continuation = 1.73; 95% CI 1.20 to 2.51). The relapse rates at the end of the continuation phase (6 months after randomization) were 35.2% for the fluoxetine group and 61.8% for the placebo group; after 1 year, they were 45.9% for the fluoxetine group and 72.0% for the placebo group.

Mean (SD) residual insomnia scores among during the baseline visit of the double-blind phase were as follows: early insomnia 0.46 (0.77), middle insomnia 0.54 (0.75), late insomnia 0.50 (0.74), total insomnia 1.50 (1.68). Mean (SD) severity of residual hypersomnia scores among during the baseline visit of the double-blind phase were as follows: early bedtime 0.16 (0.45), oversleeping 0.22 (0.49), daytime napping 0.34 (0.59), total hypersomnia 0.71 (1.00).

The severities of early (hazard ratio = 0.80; *P *= 0.19), middle (hazard ratio = 1.00; *P *= 0.99), late (hazard ratio = 0.99; *P *= 0.95), and total insomnia (hazard ratio = 0.95; *P *= 0.53) were not found to predict risk of relapse. Similarly, the severities of early bedtime (hazard ratio = 1.19; *P *= 0.44), oversleeping (hazard ratio = 1.23; *P *= 0.30), daytime napping (hazard ratio = 1.10; *P *= 0.52), and total hypersomnia (hazard ratio = 1.10; *P *= 0.34) were not found to predict risk of relapse.

## Conclusions

The present study is the first to specifically focus on testing the effects of residual sleep disturbance, including insomnia and hypersomnia, as a predictor of relapse during the continuation and maintenance treatment of MDD with the SSRI fluoxetine or placebo. In addition, although several studies have explored the relationship between clinical presentation during recovery from a major depressive episode and the risk of subsequent relapse, the present study is the first to be specifically designed to identify predictors of relapse in MDD. In summary, the results of the present analysis do not suggest that a greater burden of residual sleep disturbance is associated with a higher risk of relapse among fluoxetine responders with MDD during the continuation/maintenance phase of treatment with either fluoxetine or placebo.

While the above findings are quite interesting, several limitations to this study need to be considered in interpreting the results. Clinical trials, including the present one, typically involve a number of inclusion and exclusion criteria, and it is therefore not possible to extend findings from clinical trials to patient populations typically excluded form clinical trials (that is, patients who are actively suicidal, with psychotic symptoms, with uncontrolled medical illness, or with bipolar disorder). In addition, although the present trial did not identify a robust relationship between residual sleep disturbance and time to depressive relapse, it may have been underpowered to identify weaker effects of such residual symptoms on long-term treatment outcome. Therefore, the present results are, merely, preliminary/suggestive and need to be confirmed by future studies. Finally, the present study did not employ a second, 'active' treatment arm. It would have been interesting to explore whether or not a similar relationship between specific residual insomnia and risk of relapse would also hold for antidepressants that employ a different mechanism of action. In addition, the dependence of MDD residual symptoms on other factors such as caffeine intake and diurnal variation of depressive symptoms (such data was not available from the original study for us to analyze) would be interesting to include in future research. Ultimately, the exploration of neurobiological markers of MDD including residual symptoms will be essential to advance our knowledge of this disabling condition, which will lead to the development of new diagnostic classification, treatment and prevention.

In conclusion, the present study did not identify the severity of residual sleep disturbance among fluoxetine responders to predict a higher risk of MDD relapse. The size of our sample may have precluded us from identifying more modest effects of residual sleep disturbance on the risk of relapse in MDD patients. Future studies are needed to further explore the relationship between residual sleep disturbance and relapse in MDD.

## Competing interests

HY, LSY, SC, SJY, YC, AC, and LB declare that they have no competing interests. PJM has received research support from the National Institute of Mental Health, National Institute on Alcohol Abuse and Alcoholism, New York State Department of Mental Hygiene, NARSAD, Research Foundation for Mental Hygiene (New York State), GlaxoSmithKline, Eli Lilly, Organon and Lipha Pharmaceuticals, and has worked in an advisory/consulting capacity for GlaxoSmithKline, Somerset Pharmaceuticals, Novartis Pharmaceuticals (2008), Sanofi Aventis (2007) and Roche (2008), MF has received research support from Abbott Laboratories, Alkermes, Aspect Medical Systems, Astra-Zeneca, Bristol-Myers Squibb Company, Cephalon, Eli Lilly & Company, Forest Pharmaceuticals Inc., GlaxoSmithKline, J & J Pharmaceuticals, Lichtwer Pharma GmbH, Lorex Pharmaceuticals, Novartis, Organon Inc., PamLab, LLC, Pfizer Inc, Pharmavite, Roche, Sanofi Aventis, Solvay Pharmaceuticals, Inc. and Synthelabo, Wyeth-Ayerst Laboratories, has worked in an advisory/consulting capacity for Abbott Laboratories, Amarin, Aspect Medical Systems, Astra-Zeneca, Auspex Pharmaceuticals, Bayer AG, Best Practice Project Management, Inc., Biovail Pharmaceuticals, Inc., BrainCells, Inc. Bristol-Myers Squibb Company, Cephalon, CNS Response, Compellis, Cypress Pharmaceuticals, Dov Pharmaceuticals, Eli Lilly & Company, EPIX Pharmaceuticals, Fabre-Kramer Pharmaceuticals, Inc., Forest Pharmaceuticals Inc., GlaxoSmithKline, Grunenthal GmBH, Janssen Pharmaceutica, Jazz Pharmaceuticals, J & J Pharmaceuticals, Knoll Pharmaceutical Company, Lorex Pharmaceuticals, Lundbeck, MedAvante, Inc., Merck, Neuronetics, Novartis, Nutrition 21, Organon Inc., PamLab, LLC, Pfizer Inc, PharmaStar, Pharmavite, Precision Human Biolaboratory, Roche, Sanofi Aventis, Sepracor, Solvay Pharmaceuticals, Inc., Somaxon, Somerset Pharmaceuticals, Synthelabo, Takeda, Tetragenex, Transcept Pharmaceuticals, Vanda Pharmaceuticals Inc, Wyeth-Ayerst Laboratories, has been a speaker for Astra-Zeneca, Boehringer-Ingelheim, Bristol-Myers Squibb Company, Cephalon, Eli Lilly & Company, Forest Pharmaceuticals Inc., GlaxoSmithKline, Novartis, Organon Inc., Pfizer Inc, PharmaStar, Primedia, Reed-Elsevier and Wyeth-Ayerst Laboratories, has equity holdings in Compellis and MedAvante, and has the following royalties/patents or other income: patent applications for SPCD and for a combination of azapirones and bupropion in MDD, copyright royalties for the MGH CPFQ, DESS, and SAFER. GIP has served as a consultant to Bristol-Myers Squibb, Eli Lilly, GlaxoSmithKline, Evotec, Inflabloc Pharmaceuticals, Jazz Pharmaceuticals, Pamlab, Pfizer, Pierre Fabre, Shire and Wyeth, has received honoraria from Bristol-Myers Squibb, Eli Lilly, Evotec, GlaxoSmithKline, Inflabloc Pharmaceuticals, Jazz Pharmaceuticals, Lundbeck, Pamlab, Pfizer, Pierre Fabre, Shire, Titan Pharmaceuticals and Wyeth, has received research support from Bristol-Myers Squibb, Forest, National Institute of Mental Health, Pamlab, Pfizer and Precision Human Biolaboratories, and has served on the speakers bureau for Bristol-Myers Squibb and Pfizer.

## Authors' contributions

HY conceptualized the study, participated in the preparation of the manuscript and provided critical review of the manuscript. LSY participated in the editing of the manuscript. SC contributed in the literature search and in the preparation of the manuscript. SJY contributed in the review of the manuscript. YC was the statistician for the original study, and helped interpret the data for the current study. AC and LB conducted the statistical analysis. PJM and MF were the principal investigators of the original study and provided critical review of the study. GIP oversaw the whole project from study design to data interpretation as well as manuscript revision
